# COVID-19 treatment corticosteroids-induced mania

**DOI:** 10.1192/j.eurpsy.2022.1336

**Published:** 2022-09-01

**Authors:** J. Galvañ, I. Angélico

**Affiliations:** 1 Hospital General Universitario Gregorio Marañón, Psychiatry, Madrid, Spain; 2 Hospital Universitario Son Espases, Psychiatry, Palma de Mallorca, Spain

**Keywords:** mania, Treatment, covid, corticosteroids

## Abstract

**Introduction:**

Psychiatric disturbances induced by substances are registered in both CIE-10 and DSM-5. It is also well known, since many years, the association between mania and corticosteroids (more than 200 results in PubMed found), recently widely used during the last pandemic against COVID-19.

**Objectives:**

To remember and to point out the association of substance-induced mental disorders, warning about the experimentation in new clinical settings and raising awareness to prevent or treat its possible consequences in mental health.

**Methods:**

A two cases clinical series with COVID-19 pneumonia treated with high-doses intravenous corticosteroids during more than a week. Two women, after theirs 50s, with no personal or family psychiatric history, developing after finishing the hospital treatment, insomnia, motor and behavioral hyperactivity and dysphoric mood with irritability, but preserving clinical insight.

**Results:**

At first, these states were assessed by internists and psychologists as reactive stress anxiety and were treated with benzodiazepines and psychotherapy, without success, during more than two weeks. After a psychiatric evaluation, considering the medical history and recent use of corticosteroids, the hipomania diagnosis was pointed out. Antipsychotic treatment (low doses olanzapine chosen) was induced with total remission of symptoms in less than 15 days with *restitutio ad integrum.* Regarding these cases, an updated bibliographic review on corticosteroid-induced mania and its treatment was carried out.

**Conclusions:**

With this presentation, the authors would like to highlight, in these times of pandemic, the importance of remembering the influence and relationship of drugs use in major psychiatric syndromes, both in the causal origin and in the treatment.

**Disclosure:**

No significant relationships.

